# A Review of Major Compounds in Bilberry (*Vaccinium myrtillus* L.) Fruits and Leaves: Isolation, Purification, and Their Antiaging Effects

**DOI:** 10.3390/nu18020350

**Published:** 2026-01-21

**Authors:** Jayanta Kumar Patra, Han-Seung Shin, Gitishree Das

**Affiliations:** Department of Food Science and Biotechnology, College of Life Science and Biotechnology, Dongguk University-Seoul, Goyang-si 10326, Republic of Korea

**Keywords:** antiaging effect, bilberry fruits, bilberry leaves, bilberry compounds, isolation, purification

## Abstract

The bilberry is a low-growing plant native to northern Europe. It belongs to the genus Vaccinium. Bilberry is essential in the local diets of some countries and is used as an herbal medicine to manage several ailments. Still, it is not used for commercial farming in many countries. It has recently been known as a great source of naturally available bioactive compounds and colorants. Bilberry is a therapeutic fruit acknowledged for its rich flavonoids, anthocyanins, carotenoids, ascorbic acid, phenolic acid, tocopherols, and vitamin content. It is one of the richest sources of natural anthocyanins. The polyphenolic compounds in bilberry provide abundant antioxidant content, which are supposed to be the vital bioactive compounds accountable for various health benefits. Even though bilberry is mostly promoted for eye care or vision improvement. It is also stated to promote antioxidant defense and lower oxidative stress, having antiaging, anti-inflammatory, lipid-lowering, antimicrobial effects, lowering blood glucose and other age-related diseases, etc. Reports suggest that apart from the fruit, the leaves of bilberry are equally rich in numerous bioactive compounds of medicinal importance. This current review offers valuable insights on bilberry fruits, leaves, and extracts, providing an inclusive assessment of their bioactive compound configuration, related biological prospects, and the extraction methodology of their major compounds. This review offers a summary of the existing information on the antiaging potential of bilberry fruits and leaves, and analytically reviews the outcome of clinical trials, with special attention towards its medicinal properties.

## 1. Introduction

**Figure 1:**
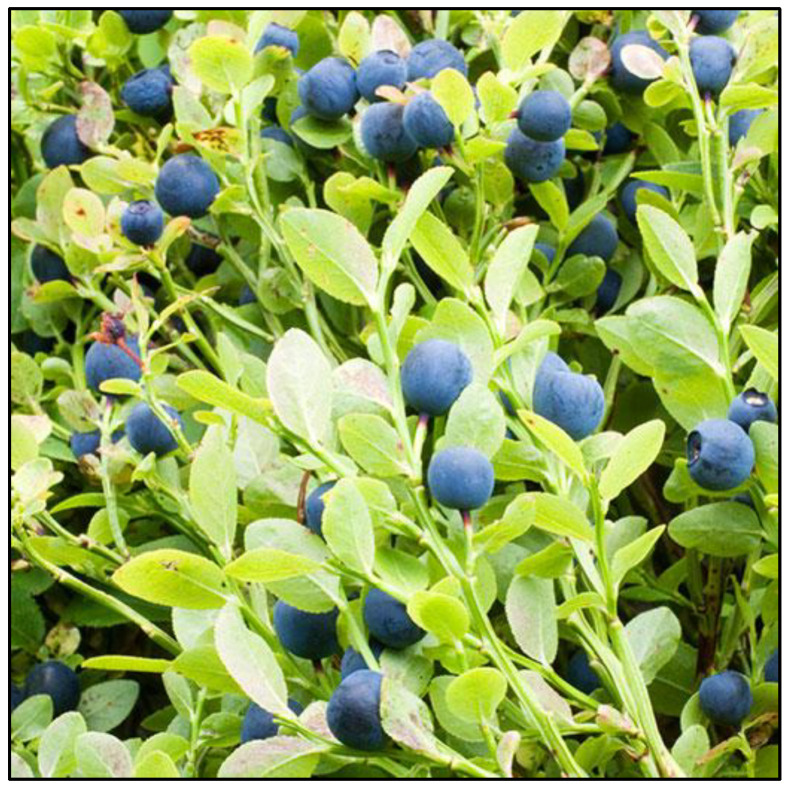
The bilberry plant showing its fruits and leaves (Source: https://www.nccih.nih.gov/health/bilberry, accessed on 15 October 2025).

The bilberry (*Vaccinium myrtillus* L.) plant of the Ericaceae family (Figure 1) is native to northern Europe and grows in a subtropical climate [1]. It is bluish black in color, 5–9 mm in diameter, and has a small fruit with a lot of seeds [2]. In addition, bog bilberries (*Vaccinium uliginosum*) and lingonberries, sometimes called red bilberries (*Vaccinium vitisidaea*), are also found in Europe [3,4,5,6]. Bilberry fruits have a high commercial significance, as they are used in generally fresh, dried, and processed foodstuffs like juice, jams, and also incorporated in food supplements [2,7]. Bilberry fruit is eaten domestically in several countries, both fresh and in extract form. It has numerous potential uses in the medicinal, cosmetics, and food sector industries [8]. The international market for bilberry fruit and its products is predicted to grow at a compound annual growth rate of 13% in the 2025–2034 forecast period [9,10]. The cultivated blueberry (*Vaccinium* sp.) fruit and the wild bilberry fruit bear a resemblance to each other in their form and taste [10]. It contains a remarkable quantity of biologically active compounds, such as quinic, malic, and citric (organic acids), as well as phenolic compounds such as delphinidin-3-O-galactoside (anthocyanins), delphinidin 3-O-glucoside (anthocyanins), flavonoids, myricetin, quercetin, kaempferol (flavonols), and chlorogenic acid (hydroxycinnamic acids), etc. [2,11].

The bilberry fruit is a rich source of minerals, fibers, and vitamins, with more than 80% water content. It also contains high levels of anthocyanins, flavonoids, polyphenols, and additional components, which display substantial biological activities. It is also associated with antioxidant ability, which can inhibit or slow down oxidative processes. It is a major natural source of anthocyanins and water-soluble flavonoids. Its fruits contain fifteen major anthocyanins comprising five anthocyanidins, aglycones delphinidin, petunidin, malvidin, peonidin, and cyanidin, which are formed as 3-O-glycosides attached by arabinose, glucose, and galactose [12]. Bilberry leaves are a rich source of secondary metabolites, containing especially polyphenolic compounds, with a variety of therapeutic effects such as being antioxidant, antibacterial, antidiabetic, anticancer, hypolipidemic, and anti-inflammatory [13,14,15,16,17,18]. It is said that bilberry leaves do not contain any toxic compounds per se; however, since they also contain tannin, precautions need to be taken for its consumption by children and the elderly, since tannin can prevent the adsorption of nutrients and reduce digestibility due to its nature of forming complexes with protein and proteases [18,19].

Aging is a complex biological process in which functional and physical changes take place in a living being over time [20]. The natural function of the human body degenerates with age, as aging is a common and natural process that happens in many tissues and organs of the body with each passing day. Furthermore, the aging effects are naturally reflected in many physical and behavioral characteristics, such as life span, reproductive ability, skin type, and workout energy, etc. These entire aging-related instances are associated with the antioxidant free radicals, due to the oxidation process [21,22]. Oxidative damage is an oxidative stress, which starts when there is an imbalance between oxidation and antioxidant mechanisms. Reactive oxygen species are an active contributor to the course of aging [22,23].

The correlation between aging and food intake is exciting, which has attained global attention. To delay the course of aging and advance the quality of life, it is necessary to add healthy food and dietary antioxidants to everyday life [20]. To support skin youthfulness for positive aging, the use of dietary supplements is also part of the scheme. In separating and defending the human body from the atmosphere, which contains physical factors, chemical agents, and pathogenic microbes, the skin membrane is a complex multilayer body part that carries out vital functions in inhibiting higher water loss, maintaining electrolyte homeostasis, thermoregulation, and immune response, etc. The aging of skin is a part of the normal process of aging, driven by chronological, inherited, and extrinsic influences [24,25]. Various healthy diets, like fruits, are a great source of natural bioactive compounds and dietary nutrients, which have antioxidant effects and help in preventing or delaying aging, including preventing additional age-related health conditions [20]. Health benefits associated with the healthy intake of fruits have drawn huge attention. The advantages of fruit intake have been documented by a large number of investigations and acknowledged as it suppresses the development of free radicals that further decreases the oxidative stress produced in the living body and defends against various types of ailments, helping to prevent aging [20]. As per the WHO (World Health Organization)’s earlier report, the inhabitants over 60 years of age around the globe will double from 11% to 22% between the year 2000–2050 [26,27]. Consequently, several studies are dedicated to upholding a healthy life by avoiding or delaying the progression of aging [27]. Although much research has mostly promoted bilberry for improving vision or sight care. Scientific evidence shows that bilberry is rich in several bioactive compounds, including polyphenols and flavonols, which have promising pharmacological importance, including antiaging effects. Compounds like anthocyanins and proanthocyanidins are shown to have positive effects against skin pigmentation, oxidative stress, and inflammation [28,29]. In addition, a few studies on the antiaging effects of bilberry fruits were also reported earlier [8,24,30,31]. Considering the presence of several bioactive compounds and their promising health-beneficial properties, the antiaging effect of bilberry has been explored in the current review, with emphasis on the major bioactive compounds along with their process of isolation, extraction, and purification methods. This review recapitulates the existing awareness on the phytochemical perspective and therapeutic potential of bilberry (fruits, leaves, and other parts), followed by the investigation of the background of scientific data related to its health claims with a distinctive emphasis on clinical results.

## 2. Methodology

A wide-ranging search, especially over the last 10 years, was performed using different web searches like Web of Science, Google Scholar, PubMed, Scopus, Science Direct, etc., to find out the systematic scientific research on bilberry fruits and leaves for their major bioactive compounds, the extraction method of major bioactive compounds, and their antiaging and health-promoting properties, etc. Keywords like bilberry, antiaging effects, and major bioactive compounds were used to search the articles. Date limits were not applied to the available scientific literature. Articles published in the English language were considered.

## 3. Bilberry: Botany, Nutritional Values, and Health Benefits

### 3.1. Nutritional Components

Bilberry (*Vaccinium myrtillus* L.), also called blue whortleberry, is a European berry species that grows wild in the northern parts of the world [3,10]. Typically, it grows in wet boreal jungles, in large quantities from the west coast of Northern Europe to the northern Asia Pacific coast [32]. It can be found in dry highland jungles, heaths, and mountains in Europe [33]. Bilberry is grouped within the Myrtillus section of the family Ericaceae. Common bilberry is a small, 30–50 cm, densely branched bushy plant. The bilberry plant bears persistent shoots and hibernating buds towards the ground soil. It is a chamaephytic plant, and cultivates on humus, rocky, acidic, and wet soils. It spread out through the dispersion of seed [32]. It has vertical green triangular branches. Its leaves are long (1–3 cm), deciduous, with a small petiole, and bright green at maturity. Its flowering period is April–June. The flowers rise in the axils of the leaves; they are not clearly marked since they are hidden or out of sight below the leaves, with five petals and five sepals. The fruit is 5–10 mm wide and dark bluish in color—a round berry. The taste is sweet [32,34]. Bilberries are a rich source of carbohydrates, containing 14.7 g of sugar/100 g of fruit [35]. It also provides additional nutritional fiber (3.6 g of dietary fiber), which can improve digestive health. Bilberries offer greater levels of B9 vitamins, vitamin K, ascorbic acid, magnesium, and dietary fiber [35]. It has vitamins A, C, and amino acids. It contains a good level of iron and other essential minerals [35].

### 3.2. Health Benefits of Bioactive Compounds

Bilberry fruits are rich in a variety of secondary metabolites like polyphenols, anthocyanins, etc., with many health-beneficial effects. Some of the health-beneficial effects of these secondary metabolites are summarized in Table 1. After the processing of bilberries in food industries, a fruit pomace composed of berry seeds and peel (by-product) in the form of a solid press bar is formed, which is high in valuable phenolic compounds and fibers [36,37]. Due to the higher content of seeds, these pomaces are also a good source of monounsaturated and polyunsaturated fatty acids [37]. Generally, bilberries hold greater levels of total hydroxycinnamic acids, anthocyanins, trans-resveratrol, total flavonols, and total phenolics, which contribute to their high anti-inflammatory and antioxidant properties. Bilberry fruits have a higher anthocyanin content [38,39,40,41]. The anthocyanins reach up to 0.1–0.25% of the fresh fruits and leaves in bilberry [42]. In a comparative investigation by Hellstrom et al. [10], the bilberries were reported to have high anthocyanin content (29 to 65 g/Kg dry weight), compared to blueberries (4.8 to 33 g/Kg dry weight) [10]. Bilberries predominantly contain delphinidin and cyanidin glucosides [3]. It contains a higher amount of proanthocyanidins and anthocyanins compared to blueberries [3]. Bilberries are gaining attention as they are rich in anthocyanins [35]. Important phytochemicals in bilberries, such as chlorogenic acid, anthocyanins, flavonoids, and procyanidins, play a substantial role in health benefits. Anthocyanins consist of 84% of the phenolic compounds, which are accountable for antioxidant effects [35].

As a whole, bilberries are great for health, which makes them an important addition to a balanced diet. The enhanced nutritional value of bilberries due to their natural antioxidants, vitamins, and mineral contents contributes to their role in overall wellbeing and better health [3]. There is no report on the presence of allergenic compounds in bilberry. Thus, bilberry can be added as a tremendous source of functional food in the diet. Bilberry is among the major sources of natural anthocyanins; these are polyphenols that include glycoside pigments (soluble), creating the purple color in vegetables, flowers, and fruits.

## 4. Major Bioactive Compounds in Bilberry

Secondary metabolites are small organic intermediate compounds originating from primary metabolites during plant stress or for protecting the plant species from adverse environmental conditions [58,59]. These compounds (phenolics, alkaloids, terpenoids, flavonoids, etc.) are not essential for the plant itself for its basic growth and development but are necessary for plant defense and these are rich in bioactive components with several medicinal properties [58,59,60]. The major bioactive compounds and their medicinal potentials in bilberry are discussed below.

### 4.1. Structure of Bioactive Compounds Found in Bilberry Fruits and Leaves

**Figure 2:**
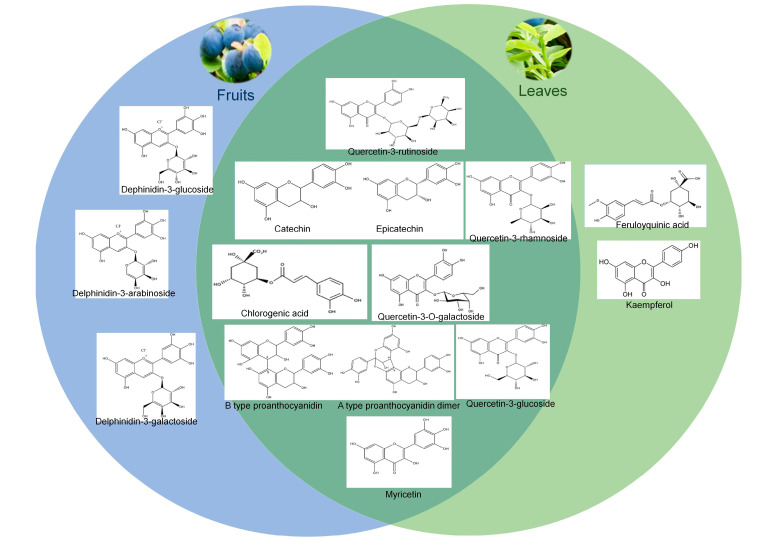
Major bioactive compounds found in the fruits and leaves of bilberry.

Studies on bilberry have shown that most of the compounds that are present in the fruit are also found in the leaves; however, their amount varies [49]. For example, leaves of bilberry contain fewer anthocyanins than fruits, but the content of phenolic compounds is higher in leaves than in fruits [61,62]. The major group of compounds in bilberry fruits mainly belongs to polyphenols such as phenolic acids, anthocyanins, flavonols, and flavan-3-ols (Figure 2) [63,64]. In addition, other compounds such as trans-resveratrol, flavonoids, like catechins and proanthocyanidins (flavan-3-ols), kaempferol, quercetin, myricetin (flavonols), hydroxycinnamic and hydroxybenzoic acids (phenolic acids), and derivatives of stilbenes, are also found in bilberries [3,61,65,66]. The key compounds documented in *V. myrtillus* fruits are anthocyanins and flavonols, kaempferol, quercetin, and myricetin glycosides, isorhamnetin glycosides, syringetin, and laricitrin, etc. [64,67]. The main chemical compounds found in bilberries, which are responsible for antioxidant and antiaging effects, are hydroxycinnamic acids, flavonols, phenolics, and trans-resveratrol, etc. [3]. A few research studies revealed the existence of stilbenes in bilberry fruits. Anthocyanins are considered highly important natural compounds in bilberry [10]. As per the numerous studies described in *V. myrtillus* fruits, anthocyanins are the most abundant polyphenol, which is accountable for the blue (dark) characteristic of bilberry. Bilberry anthocyanins are mostly conjugated with one or more sugar deposits connected through hydroxyl groups of aromatic core [41,68]. They are categorized by 15 anthocyanidin heterosides that come from the mixture of five anthocyanins, including anthocyanidins like cyanidin, delphinidin, malvidin, petunidin, and peonidin, with three types of sugars such as arabinose, galactose, and glucose [10,41,68,69]. The polyphenolic elements have high antioxidants, which are supposed to be the important bioactive compounds accountable for various stated health benefits of intaking berries. Epidemiologic research proposes that the intake of anthocyanins decreases the threat of diabetes, vision diseases, cardiovascular disease, arthritis, and cancer, owing to their antioxidant, anti-inflammatory, and antiaging properties [3,70]. Usually, the nutraceutical potential of most of the berries is connected with their abundant polyphenol content, especially the anthocyanins [10]. Bilberry is exceptionally rich in anthocyanins, which are present all over the body; however, anthocyanins in blueberries are present primarily in the skin [10,71].

The content of polyphenols could be one of the conditions for estimating the biological prospective of natural plant resources like bilberry. Mainly, flavonoids and anthocyanins are the major plant phenolic compounds, which are also found in bilberry in higher amounts [30]. The anthocyanin content is three to four times greater in frozen bilberry compared to fresh blueberry [30]. In addition, hydroxycinnamic acids, flavonolignans, flavanols, flavonols, and a little of anthocyanins, triterpenes, and phytosterols are also found in the leaves of bilberry (Figure 2) [18,57]. Several reports confirm that hydroxycinnamic acids, cinchonains, flavonols, proanthocyanidins, and iridoids are found in the bilberry leaves [51,72]. As of now, about 70 compounds have been identified from the bilberry leaves, and among them, hydroxycinnamic acids are the major compounds based on LC/MS qualitative and quantitative analysis studies [18,73]. The general group of hydroxycinnamic acid concentration is higher in the bilberry leaves than in its fruit [71]. In the leaves of bilberry, sufficient caffeoyl-shikimic, feroylquinic acid, chlorogenic acid, its isomers, and a small amount of caffeic acid are found [73,74]. Chlorogenic acid, isoquercetin, and resveratrol are reported to be the most abundant phenolic compounds found in bilberry leaf extract [14]. Another important group of phenolics is flavonoids present in the bilberry leaves. The Quercetin-3-O-glucuronide is a plentiful flavonol that exists in bilberry leaves [73]. The other flavonols in bilberry leaves are quercetin-3-O-(4′′-HMG)-α-rhamnoside, quercetin-3-O-β-galactoside, quercetin-3-O-glucoside, quercetin, and quercetin-3-O-arabinoside, including three kaempferol glycosides [73,75]. Additionally, lavan-3-ols, six different isomers of two coumaroyl iridoids, three proanthocyanidins, and cinchonain, etc., are also detected in the leaves [76,77]. Among triterpenic acids, oleanolic and ursolic acids are found in high concentrations in the bilberry leaves when organic solvents are used for the extraction of compounds from leaves [78]. In addition, palmitic acid, linoleic acid, and linolenic acid are also found in bilberry leaves [79]. Brasanac–Vukanovic et al. [57] detected Pyrogallol only in the leaves of bilberry.

### 4.2. Extraction and Identification of Major Compounds from Bilberry Fruit and Leaves

#### 4.2.1. Extraction of Major Compounds from Bilberry Fruit and Leaves

Sample preparation and extraction are the first steps in the identification of any bioactive compound for berries [80]. The different extraction techniques adopted can significantly affect the type of compounds and their concentration [81]. Conventional extraction procedures, such as maceration and solvent extraction procedures, were adopted; later on, advanced technology, such as ultrasound-assisted extraction and supercritical fluid extraction procedures, was used for better output [81]. The performance of the extraction procedure greatly depends on factors like the type of solvent used, the amount of temperature, pressure, and time adopted, etc. [80]. The specific extraction procedure was selected on the basis of the targeted compound and its quantity required [82]. The extraction of major secondary metabolites from the bilberry fruits and leaves is reported to be performed by using a variety of extraction procedures such as classical solvent extraction, microwave-assisted extraction, solvent maceration, ultrasound-assisted extraction, crude extraction method with deoxygenated methanol (flushed by nitrogen for 5 min), homogenization, infusion, soxhlet extraction, etc. [12,48,56,57,71,74,83,84]. The different types of extraction techniques used by various authors for the extraction of major secondary metabolites from bilberry fruits and leaves are summarized in Table 2 and Table 3. For example, for the extraction of major secondary metabolites, like anthocyanins, hydroxycinnamic acids, flavonols, etc., from the bilberry leaves, fruits, and rhizome, the frozen maceration in acidified methanol process was adopted by Riihinen et al. [71]. After grinding the frozen samples in liquid nitrogen and macerating with acidified methanol, the samples were subjected for identification of the compounds.

#### 4.2.2. Identification of Major Compounds from Bilberry Fruit and Leaves

The complex nature of berries and their products requires the most advanced analytical technology for qualitative and quantitative analysis [80]. Usually, chromatographic separation techniques are adopted for better identification; however, there are several challenges due to the complex nature and existence of intrusive constituents that can interfere with the compound identification process. A major challenge is the sample preparation techniques, which affect the quality of the result significantly, and thus proper selection of the extraction procedure and the corresponding analytical techniques for identification of specific compounds are utmost important [80]. For the identification of major compounds from bilberry fruits and leaves, various standard analytical techniques, such as the Folin–Ciocalteau method (spectrophotometric), spectrophotometric pH differential method, HPLC with a UV/VIS detector, HPLC–mass spectrometry analysis, liquid chromatography tandem mass spectrometry, ultra-HPLC system with tandem mass spectrometry, using heated electrospray ionization, etc., are used [48,56,57,71,83,87]. Anthocyanin-rich berries are hard to analyze due to the similar profile of their bioactive compounds [90]. Hence, more advanced techniques such as HPLC-MS/MS are used for high sensitivity and precision [90,91]. Furthermore, UV/Vis spectroscopy, a well-known method, is usually used for phytochemical analysis such as total phenol content, total flavonoid content, etc. [92]. A detailed list of analytical techniques used for the identification of specific compounds is summarized in Table 2 and Table 3. For instance, for the identification of anthocyanins, hydroxycinnamic acids, flavonols, etc., from the bilberry leaves, fruits, and rhizome, HPLC with diode array detection, and UV–vis spectral analysis was used. Before HPLC, the extracts were filtered and analyzed by using a LiChroCART Purospher RP-18e column [71]. Diode array detection was used for UV–vis spectral analysis, and quantification of the individual compounds was performed within the linear range using standard curves of representative compounds [71]. Characterization and identification of anthocyanins present in bilberry was performed by HPLC with a UV/VIS detector [48]. For identifying trans-resveratrol from bilberry, LC-MS/MS analysis was adopted [84].

## 5. Bioactivity of Bilberry

### 5.1. Antiaging Effect of Bilberry and Possible Mode of Action of Its Bioactive Compounds

Evidence from scientific literature showed that bilberry possesses many pharmacological properties, such as antiaging, antioxidant, antidiabetic, antiviral, antimicrobial, anticancer, hypolipidemic, and anti-inflammatory properties, which are correlated to the existence of several bioactive compounds in bilberry [18]. Aging is a common and normal process that takes place in different tissues and body parts of living organisms [22]. With the passing of time and age, the natural functions of the body deteriorate. The aging special effects can be found in several behavioral aspects and characteristics, like life time, skin texture, body strength, bone strength, reproductive ability, eyesight, exercise vitality, etc. [21,22]. Different studies have shown that the process of aging is supposed to create an imbalance between antioxidative defense and oxidative damage [22]. Thus, inhibiting oxidative damage by increasing the antioxidative resistance might neutralize aging and age-associated complaints [93]. The antioxidant signaling pathway of nuclear factor erythroid 2-like 2-transcription factor with high sensitivity to oxidative stress is believed to be the most promising antioxidant defense mechanism against oxidative stress [94]. This transcription factor can bind to the androgen response element in the nucleus and boost the transcription of several antioxidant genes like catalase, glutathione peroxidase, and superoxide dismutase, and remove the excess amount of reactive oxygen species [22].

Through the reduction in oxidative stress influenced by extreme reactive oxygen species, anthocyanins, which are well known as an antioxidant compound, could delay the aging process, thereby attracting considerable attention from researchers around the world. Additionally, UV radiation might also prompt oxidative stress by forming reactive oxygen species, which could speed up the aging process [22,95]. As per the earlier report, the bilberry could influence the peroxide and antioxidant enzymes. Some reports suggest that anthocyanins (freeze-dried) are capable of binding free radicals precisely to encourage antioxidant potential [96]. In addition, indirect antioxidant potential can also be attained by dropping extra oxidants that have already oxidized [22,96]. Moreover, through numerous approaches, anthocyanins can directly activate the antioxidant enzyme system, through instigating the superoxide dismutase production and activity, catalase activity, and by strengthening the endogenous antioxidant system [97]. Anthocyanins are also able to directly bind to malondialdehyde and decrease the amount of malondialdehyde content [98]. In the earlier research, it was concluded that the bilberry could evidently ease the aging process of UV-treated and natural male *Drosophila melanogaster*, along with enhanced fertility and extended life span [22]. Moreover, the mechanisms associated with it specified that, the anthocyanin extracts from bilberry could efficiently extend the average natural lifespan, increase the ability of reproduction and increase the antioxidant potential in the regular as well as the UV-treated flies. Principally, the anthocyanin extracts from bilberries can modify the growth cycle, decreasing the antioxidant expression levels, sex ratio, and autophagy-associated genes in the UV-treated flies and in offspring [22]. Collectively, the outcome of research proved that the anthocyanin extracts from bilberry supplementation can efficiently alleviate the process of aging in the *D. melanogaster* body [22].

The bioactive compound from bilberry, like anthocyanins, is among the richest sources of polyphenols [22]. These are extensively utilized in the cosmetic, food, and medicine industries owing to their antioxidant properties. Proanthocyanidin-rich foods are reported to be effective against skin pigmentation [28]. In addition, anthocyanins have also been reported to be potentially efficient in protecting the skin against oxidative stress and inflammation [29]. Very few studies have studied the bilberry fruit’s antiaging effect and its mechanism of action. Multiple mechanisms of action for these bioactive compounds exhibiting potential activities are discussed in a series of literature (Table 4). As per an earlier research result, the effectiveness of fermented bilberry extract and its oral supplementation clearly displayed promising skin aging mark reduction, improvement in the complexion, and firmness of skin in postmenopausal women, and in the general population [24]. The supposed machinery causing declines in skin aging signs can be associated with an upsurge in the skin’s antioxidant capability and a decline in skin inflammation [24]. The outcome of the said study was attributed to the bioavailability of polyphenolic components in bilberry extract, which leads to its distribution at the skin level [24]. Earlier studies on people have also stated the beneficial impact of bilberry in decreasing inflammation by means of down-regulating the expression of pro-inflammatory cytokines and enzymes, modifying the signaling pathways, and decreasing the level of ROS [24,99]. Furthermore, anthocyanins and proanthocyanidins (polyphenols) can influence skin wellbeing through progressive communications with gut flora [24].

In a study, the authors have studied the antiaging potential of chitosan-based hydrogel supplemented with bilberry fruit extract and concluded that *V. myrtillus* and *V. corymbosum* dry fruit extracts rich hydrogels, displaying promising antiaging effects [30]. In another study, the author examined the UV protective and inhibitory properties of bilberry fruit and leaf extracts against skin conditioning- and skin aging-related enzymes such as tyrosinase, hyaluronidase, and collagenase [8]. The author concluded that the natural deep eutectic solvent extracts of both the fruits and leaves are highly effective against these enzymes, with higher results from the leaf extracts as compared to the fruit extracts [8]. The extracts with the best activities also displayed a good safety profile in a 24 h in vivo study on human volunteers [8]. Furthermore, a patent (JP2010531816A) was filed on the cosmetic-related properties of bilberry extract-containing anthocyanins, and the author has claimed that compared with other conventional beauty products, the bilberry extract of the said invention has a strong effect and high safety [31]. The health-promoting effects of bilberry anthocyanin on healthy aging were investigated using 12-month-old, aging female Sprague Dawley rats in a study. And the author concluded that bilberry anthocyanin consumption has an important function in diminishing aging-induced oxidative stress and reducing the permeability of the intestinal epithelial barrier through induction of protective autophagy, thus promoting healthy aging in female rats [100]. Here, the author has shown that the middle dose of the bilberry anthocyanin was able to induce phosphorylation of AMP-activated protein kinase (AMPK) and Forkhead box O3a (FOXO3a) and inhibited the phosphorylation of Mammalian target of rapamycin (mTOR), which showed that bilberry anthocyanin could induce autophagy through the AMPK–mTOR signaling pathways [100]. Age-related macular degeneration is a main cause of vision loss in the elderly, substantially lessening quality of life, and preclinical studies suggest that extracts from various berries, such as bilberry, can enhance retinal health by reducing oxidative stress and inflammation [43]. Studies have suggested that anthocyanidins, including cyanidin, which is abundantly present in bilberry, may play vital roles in reducing the risk of many age-related diseases [101]. A study by Bohn et al. [102] suggested that a nine-week bilberry/red grape juice intervention was able to decrease the level of biomarkers of inflammation and tissue damage in aged men (age ≥ 67 years). It is said that with increasing age, there is an increased risk for women to develop perimenopause syndrome that is harmful to women’s physical and mental health and in this context, a research group has studied the health-beneficial effects of bilberry anthocyanin on aging perimenopausal Sprague Dawley rats and found that bilberry anthocyanin has a great effect on enhancing the serum cholesterol in natural aging perimenopausal rats via the estrogen receptor signaling pathway [103]. Bilberry could enhance the clearance of beta-amyloid deposits in drusen, a characteristic feature of age-related macular degeneration, and hinder the activation of STAT3 and NF-κB, the pathways related to inflammation and cell survival [104,105]. Anthocyanins in bilberry are reported to protect mitochondrial function by significantly reducing the beta-amyloid protein clump load and Amyloid-beta 42 levels, which mostly decline with age [106].

Bilberry extract’s antiaging effect, primarily from its effective antioxidant and anti-inflammatory properties, is mainly owing to its high content of anthocyanins. The bioactive compounds of bilberry help in defending cells from oxidative damage, decreasing inflammation, and neutralizing free radicals, contributing to a healthier and younger-looking skin texture. In LPS-lured RAW 264.7 cells, bilberry extract can also suppress the generation of nitric oxide and reverse pro-inflammatory cytokines like COX-2, TNF-α, iNOS, and IL-6 [107]. According to the above outcomes, it is suggested that bilberry extract is a natural, powerful antiaging agent as a rich source of anthocyanins [107]. By controlling antioxidant activities, the bilberry extract might play an essential role in homeostasis. In addition, oxidative stress might trigger inflammations, and to protect the body’s cells and tissue, the immune cells, like macrophages, are activated against the antigens, foreign invasion, cell debris, pathogens, and so on [107]. In terms of diminishing the signs of skin aging, the supposed mechanism underlying this could be associated with a rise in the skin’s antioxidant ability and a decline in the inflammation of the skin [107].

**Table 4:** Summary of specific compounds in Bilberry affecting specific pathways at the molecular or cellular level in the aging process.

Specific Compounds	Pathways	References
Anthocyanins	Bilberry anthocyanin consumption has an important function in diminishing aging-induced oxidative stress and reducing the permeability of the intestinal epithelial barrier through induction of protective autophagy. The middle dose of the bilberry anthocyanin was able to induce phosphorylation of AMP-activated protein kinase (AMPK) and Forkhead box O3a (FOXO3a) and inhibited the phosphorylation of Mammalian target of rapamycin (mTOR), which showed that bilberry anthocyanin could induce autophagy through the AMPK–mTOR signaling pathways.	[100]
	Bilberry anthocyanin has a great effect on enhancing the serum cholesterol in natural aging perimenopausal rats via the estrogen receptor signaling pathway.	[103]
	Bilberry could enhance the clearance of beta-amyloid deposits in drusen, a characteristic feature of age-related macular degeneration, and hinder the activation of STAT3 and NF-κB, the pathways related to inflammation and cell survival.	[104,105]
	Anthocyanins in bilberry are reported to protect mitochondrial function by significantly reducing the beta-amyloid protein clump load and Amyloid-beta 42 levels, which mostly decline with age.	[106]
Polyphenolic compounds	Fermented bilberry extract can enhance the parameters related to skin complexion, like skin lightness, skin pigmentation, and skin color redness factor, and polyphenolic components in bilberry extract lead to its distribution at the skin level.	[24]
Bilberry compounds	The beneficial impact of bilberry lies in decreasing inflammation by means of down-regulating the expression of pro-inflammatory cytokines, enzymes, and modifying the signaling pathways, and decreasing the level of ROS.	[24,99]
Bilberry compounds	In LPS-lured RAW 264.7 cells, bilberry extract can suppress the generation of nitric oxide and reverse pro-inflammatory cytokines like COX-2, TNF-α, iNOS, and IL-6.	[107]

### 5.2. Other Miscellaneous Properties of Bilberry

Bilberry (*Vaccinium myrtillus* L.) is generally recognized as a functional food, owing to its numerous health-stimulating bioactive compounds. It is rich in vitamins, flavonoids, anthocyanins, ascorbic acid, phenolic acid, tocopherols, and carotenoids. Bilberry has anticancer, antioxidant, antimicrobial, eye-protective, neuroprotective, anti-obesity, cardiovascular effect, antidiabetic, and anti-inflammatory activities because of its enormous phytoconstituents [18,32,44,51,108,109]. In oriental medicine, bilberry has been used for a long time for the prevention and management of several ailments, like hyperglycemia, diabetes, obesity, visual acuity, inflammatory and cardiovascular diseases, cancer, and dyslipidemia, etc. The antioxidant effect of bilberry was tested in an earlier study by evaluating the capability of bilberry extracts to scavenge free radicals [30]. In another preclinical study, mouse liver, after being vulnerable to bilberry extract rich in anthocyanins, displayed a clear degeneration in the gene expression of nitric oxide synthase (iNOS), TNF-α, IL-1β, and IL-6 inflammatory markers, together with a successive lessening in the levels of iNOS, TNF-α, and NF-κB, which specified the effective defending effects of bilberry extracts against inflammation [110].

In an earlier study, the in vitro antioxidant effect of the bilberry ethanol extract was estimated as DPPH, ABTS, nitrite radical scavenging, and ferric-reducing free radical scavenging activities [107]. The results revealed a substantial antioxidant effect and inhibition of linoleic acid oxidation in a concentration-related fashion [107]. In a study, anthocyanin-rich bilberry extract administered to rats was reported to display significantly greater plasma antioxidant action than the control rats [111]. In another in vivo study on a mouse model, when the mice are given an intake diet of 0.5% bilberry extract for 14 days, the anthocyanin plasma levels increased to a maximum of 0.26 μM [112]. Two primary anthocyanins, such as malvidin-3-glucoside and malvidin-3-galactoside, were identified in the plasma. Bilberry extracts prepared using ethanol (acidified), rich in anthocyanins, showed higher antioxidant results. In addition, the proposed formulations displayed anti-inflammatory properties with marginal side effects and slight toxicity [7]. In another study, the author evaluated the anti-inflammatory properties of anthocyanin-rich bilberry extracts by inspecting their antioxidant properties and their potential effect on rat peritoneal macrophage inflammatory responses [113]. The results concluded that the bilberry extract can influence the inflammatory response by effectively persuading many molecular targets that could underlie the inflammatory response and provide anti-inflammatory effects without any adverse effects on the normal body cells [113].

## 6. Clinical Studies/Trials Related to Bilberry

Several studies on the medicinal potential of bilberry and its compounds have been undertaken so far; however, only 20 clinical trial reports have been published on the clinical trials website (https://clinicaltrials.gov/search?cond=bilberry, accessed on 22 October 2025). We have discussed these articles along with some more in this current section (Supplementary Table S1). Some clinical trials of metabolic ailments stated that eating bilberry fruits (cold, pre-treated, or garden-fresh) or as juices can decrease inflammatory markers significantly [7,99,114]. For example, bilberry juice was able to lessen some inflammatory cytokine levels, including C-reactive protein and IL-6 in the plasma, thereby controlling inflammatory response [115].

A study by Zhang and Dai [22] discusses the influence of anthocyanin extracts from bilberry on various characteristics of UV-treated and natural *D. melanogaster* (male), including their antioxidant capacity, lifespan, and fertility, alongside the mechanisms associated with them. The data specified that anthocyanin extracts from bilberry can efficiently extend average and maximum natural life span, and also increase the ability of reproduction in regular and UV-treated flies, in addition to enhancing their antioxidant capabilities. Specifically, the influence of anthocyanin extracts from bilberry considerably altered the content of ROS, sex ratio, and growth cycle in offspring, and reduced the antioxidant and autophagy-related gene expression in the UV-mediated flies [22].

According to earlier studies on bilberry, water-soluble bilberry fruit extract was able to decrease the UVA- and UVB-induced injury in a human cell line (keratinocyte). It can also decrease genotoxicity, cytotoxicity (UVB-treated), and lipid peroxidation (UVB-treated). With UVA-treated injury, bilberry can reduce genotoxicity and also the imbalance of the redox intracellular position [32]. Furthermore, a cream combined with both the extract from bilberry leaves and the oil from bilberry seeds was reported to improve the hydration of skin as per the extended clinical trial (on 25 volunteers) for one month [14,32]. Bilberry fruit extracts and fruits achieve the utmost positive clinical results in chronic inflammatory and dyslipidemia, as well as ailments like metabolic syndrome, oral ulcerative colitis, mucosa inflammation, and higher cardiovascular threat allied with higher inflammatory serum values [32].

## 7. Conclusions and Future Prospects

At present, berries have gained greater interest among the world’s population as functional food ingredients due to their health-beneficial effects and other industrial and nutraceutical uses. Fruits are a rich source of natural compounds, such as antioxidants, dietary fiber, and polyphenols, and subsequently, numerous potential health benefits are associated with their intake. Bilberry is a common dietary fruit rich in polyphenols like anthocyanins and flavonoids with enormous antioxidant potential. Furthermore, the bilberry leaves are rich in phenolic compounds like hydroxycinnamic acids, flavanols, and flavonols. A variety of extraction and analytical techniques are used for the extraction and identification of major bioactive compounds from bilberry fruits and leaves. It is essential to select appropriate extraction and analytical techniques for better efficiency. Potential health benefits associated with bilberries include antioxidative, anti-obesity, antidiabetic, anticancer, neuroprotective, hypoglycemic, vision-enhancing, and immunomodulatory activities, but limited research is available on their antiaging properties. Preclinical studies and some clinical studies have shown that bioactive compounds from bilberry can mitigate oxidative stress, diminish inflammation, improve visual function, and slow down the progression of age-related macular degeneration. The health-supporting potential of bilberry is typically linked with its high anthocyanin content. The anthocyanins are present throughout the berry; however, in the case of the blueberries, they are mostly concentrated in the skin. This property gives bilberry an upper hand over the blueberry.

Studies show that the effectiveness of bilberry extracts plays a major role in diminishing skin aging marks, refining skin complexion, and improving skin firmness in the human population. Thus, as part of a skin antiaging policy, to bridge the difference between the health span and life span, the intake of bilberries might be beneficial to make the skin look healthy. The effectiveness of oral supplementation of fermented bilberry extract is established in terms of reducing the signs of skin aging, improving skin complexion, and the firmness of skin of human beings. The supposed mechanism underlying this property is that the reductions in the skin aging signs could be associated with a rise in the antioxidant capability of skin, thereby resulting in a decline in the inflammation of skin. However, in terms of reducing skin inflammation and influencing the skin, the probable role of bilberry needs to be further examined. Subsequently, the phenolic compounds in bilberry leaves are also known as antioxidant and anti-inflammatory agents. However, the efficiency of eating berry leaves is very much lesser. Further studies on various biomarkers of ailment and the probable toxicity of bilberry fruits, leaves, and other products are essential. The nonexistence of novel clinical investigations on bilberry and its various pharmacological potentials is observed.

Bilberry fruits, fruit extracts, leaves, and other products have accomplished maximum affirmative clinical outcomes in dyslipidemias and long-lasting inflammatory ailments like oral mucosa inflammation, ulcerative colitis, metabolic syndrome, and amplified cardiovascular risk associated with elevated inflammatory serum values. However, still more research is needed to explore the antiaging effects of bilberries. This inclusive review provides valuable understandings into the major bioactive compounds, extraction effectiveness, and bioactivity of bilberry, offering perspectives for future research (e.g., recognition of the major compounds accountable for its antiaging effects) and supporting the potential uses and applications of these extracts/fruit and leaves in functional foods, pharmaceuticals, and cosmetic sectors.

## Figures and Tables

**Table 1:** Major secondary metabolites in bilberry and their health-beneficial effects.

Secondary Metabolites	Health Beneficial Effects	References
Anthocyanins	Enhances neuroprotection; decreases cardiovascular disease risk; promotes weight management; lowers type 2 diabetes risk; acts as a prebiotic; improves gut microbiota; reduces chronic inflammation; enhances vision and brain function; anti-inflammatory and antioxidant properties.	[43,44,45,46,47,48,49]
Phenolics	Robust antioxidant effects; anti-inflammatory, anti-allergic, anti-thrombotic, and anticancer properties. They help protect the skin from oxidative stress and UV damage; support cognitive function; decrease the risk of chronic diseases such as cardiovascular and neurodegenerative conditions. Bilberry pomace is a good source of polyphenolic compounds with potential antiradical and hepato-protective activities. Leaves, stems, fruits, seed oil exhibited antioxidant and antidiabetic properties.	[14,16,47,50,51,52]
Hydroxycinnamic acids	Strong antioxidants that decrease oxidative stress, dropping the risk of cardiovascular and neurodegenerative diseases, and cancer. Display anti-inflammatory and antimicrobial effects.	[3,53,54,55]
Flavonols	It has antioxidants, anti-inflammatory, anticancer, antidiabetic, antimalarial, antimicrobial, neuroprotective, cardio-protective, hepato-protective, antiviral, and antihypertensive properties.	[49,55,56,57]
Flavanols	Have antioxidant and antimicrobial properties.	[49,56,57]

**Table 2:** Major bioactive secondary metabolites from bilberry fruits and their extraction and identification procedure.

Bioactive Compounds	Quantity	Technique Used for Extraction	Technique Used for Identification	Reference
Total Anthocyanins	6102–7465 mg/100 g dry weight	Classical solvent extraction, microwave-assisted extraction, solvent maceration, ultrasound-assisted extraction.	HPLC with a UV/VIS detector; spectrophotometric pH differential method; HPLC Analysis	[12,48]
	405 mg/100 g fresh weight	Solvent maceration	Modified pH differential photometric method	[85]
	1682.37 ± 75.92 mg/L (in liqueur, from whole fruit); 1788.01 ± 42.25 mg/L (in juice)	Bilberry liqueur preparation and used after 3 months	HPLC–mass spectrometry analysis	[83]
	20,256 μg/g (peels) 1040 μg/g (pulp)	Frozen and macerated	HPLC with diode array detection and UV–vis spectral analysis	[71]
	50.00 ± 1.22 μg/L (crude extract)	Crude extraction method with deoxygenated methanol (flushed by nitrogen for 5 min)	Liquid Chromatography Tandem Mass Spectrometry (LC–MS/MS)	[47]
	1210.3 ± 111.5 mg cyanidin 3-glucoside equivalents/100 g fw	Crude extraction method with deoxygenated methanol (flushed by nitrogen for 5 min)	Liquid Chromatography Tandem Mass Spectrometry (LC–MS/MS)	[84]
	330 Cyanidin-3-O-glucoside mg/100 g (Lot 1) and 344 Cyanidin-3-O-glucoside mg/100 g (Lot 2)	homogenization	HPLC analysis	[86]
Trans-resveratrol	0. 2 mg/100 g fresh weight	Crude extraction method with deoxygenated methanol (flushed by nitrogen for 5 min)	Liquid Chromatography Tandem Mass Spectrometry (LC–MS/MS)	[84]
Total hydroxycinnamic acids	207.00 μg/g (peels), 163.00 μg/g (pulp), 10,797.00 μg/g (green leaves), 22,256.00 μg/g (red leaves) [71]; 117.04 ± 6.20 mg/L (in liqueur, from whole fruit) [83]	Frozen and macerated in acidified methanol [71]; bilberry liqueur preparation and used after 3 months [83]	HPLC with diode array detection and UV–vis spectral analysis [71]; ultra-HPLC system with tandem mass spectrometry, using heated electrospray ionization [83]	[71,83]
Total Flavonols	206 μg/g (peels), 15 μg/g (pulp), 3540 μg/g (green leaves), 10,613 μg/g (red leaves) [71]; 9.91 ± 1.24 mg/L (in liqueur, from whole fruit), 7.32 ± 1.30 mg/L (in liqueur, from juice) [83]; 1.4 mg/100 g fresh weight [84]	Frozen and macerated in acidified methanol and hydrolysed with acid for the analysis of flavonols as aglycones [71]; Bilberry liqueur preparation and used after 3 months [83]; crude extraction method with deoxygenated methanol (flushed by nitrogen for 5 min) [84]	HPLC with diode array detection and UV–vis spectral analysis [71]; ultra-HPLC system with tandem mass spectrometry, using heated electrospray ionization [83]; liquid chromatography–mass spectrometry (LC–MS) [84]	[71,83,84]
Total Phenolics	35.3 mg/100 g fresh weight [84]; 1903.45 ± 85.95 mg/L (in liqueur, from whole fruit), 2061.77 ± 55.96 mg/L (in liqueur, from juice) [83]; 577 gallic ac. mg/100 g (Lot 1) and 614 gallic ac. mg/100 g (Lot 2) [86]; 18.18 ± 0.59 μg/L (crude extract) [47]	Crude extraction method with deoxygenated methanol (flushed by nitrogen for 5 min) [84]; bilberry liqueur preparation and used after 3 months [83]; homogenization [86]; crude extraction method with deoxygenated methanol (flushed by nitrogen for 5 min) [47]	Liquid chromatography–mass spectrometry (LC–MS) [84]; ultra-HPLC system with tandem mass spectrometry, using heated electrospray ionization [83]; Folin–Ciocalteu method (spectrophotometric) [86]; Folin–Ciocalteu reagent spectrophotometric method [47]	[47,83,84,86]

**Table 3:** Different types of secondary metabolites are found in the leaves of bilberry.

Group	Compound	Concentration Range	Technique Used for Extraction	Technique Used for Identification	References
Hydroxycinnamic Acids (mg/g dry weight)	Chlorogenic acid	0.07–104.7	Maceration and infusion; ultrasound-assisted extraction	HPLC analysis; HPLC-coupled with a diode-array detector analysis; HPLC-coupled with a diode-array detector and MS-detector; HPLC-coupled with a diode-array detector and electrospray ionization mass spectrometer	[49,57,74,87]
	Ferulic acid	0.11–0.28	Infusion, maceration, Soxhlet extraction	HPLC analysis	[57]
	Feruloylquinic acid	47.66–59.65	Ultrasound-assisted extraction	HPLC-coupled with a diode-array detector and MS-detector	[49]
	Sinapic acid	0.18–0.63	Maceration and Soxhlet extraction	HPLC analysis	[57]
Hydroxybenzoic acids	Gallic acid	0.54–0.80 mg/g dry weight [57]; 6.53–352.3 mg/kg [56]	Soxhlet extraction	HPLC analysis	[56,57]
	Syringic acid	24.09–960.56 mg/kg	Soxhlet extraction	HPLC analysis	[56]
	Protocatechuic acid	1.4–1.74 mg/g	Maceration, infusion, and Soxhlet extraction	HPLC analysis	[57]
	Vanillic acid	18.00–1156.80 mg/kg	Soxhlet extraction	HPLC analysis	[56]
Other polyphenols	Pyrogallol	2.45–3.46 mg/g	Soxhlet extraction	HPLC analysis	[57]
	Resveratrol	4.60–5.15 mg/g dry weight [57]; 1.5–8.89 mg/kg [56]	Maceration, infusion, and Soxhlet extraction	HPLC analysis	[56,57]
Flavanols	Gallocatechin	4.84–15.37 mg/g	Ultrasound-assisted extraction	HPLC-coupled with a diode-array detector and MS detector	[49]
	Epigallocatechin	7.23–197.8 mg/kg	Soxhlet extraction	HPLC analysis	[56]
	Catechin	7.31–95.59 mg/kg [56]; 4.79–21.57 mg/g [49]	Ultrasound-assisted extraction, Soxhlet extraction	HPLC analysis; HPLC-coupled with a diode-array detector and MS-detector; HPLC-coupled with a diode-array detector and Electrospray Ionization Mass Spectrometer	[49,56,72,87]
	Epicatechin	2.55–84.06 mg/kg [56]; 4.31–9.66 mg/g [49]; 4.38–5.57 mg/g [57]	Maceration, infusion, Soxhlet extraction, ultrasound-assisted extraction	HPLC analysis; HPLC-coupled with a diode-array detector and MS-detector; HPLC-coupled with a diode-array detector and electrospray ionization mass spectrometer	[49,56,57,87]
Flavonols	Myricetin	49.4–237.6 mg/kg	Soxhlet extraction	HPLC Analysis	[56]
	Quercetin 3-O-rutinoside	42.34–49.83 mg/g [49]	Ultrasound-assisted extraction	HPLC-coupled with a diode-array detector and MS-detector; HPLC-coupled with a diode-array detector and electrospray ionization mass spectrometer	[49,88]
	Quercetin 3-O-glucoside	1.29–2.37 mg/g [49]	Ultrasound-assisted extraction	HPLC-coupled with a diode-array detector and MS-detector; HPLC-coupled with a diode-array detector and electrospray ionization mass spectrometer	[49,87]
	Quercetin 3-O-rhamnoside	0.11–1.65 mg/g	Maceration, ultrasound-assisted Extraction	HPLC-coupled with a diode-array detector and an electrospray ionization mass spectrometer	[87,88]
	Quercetin 3-O-galactoside (Hyperozide)	2.45 mg/g	Ultrasound-assisted extraction	HPLC-coupled with a diode-array detector and an Electrospray Ionization Mass Spectrometer	[88]
	Quercetin	0.99–11.83 mg/kg [56]; 1.16–3.69 mg/g [49]; 1.16–7.27 mg/g [57]	Maceration, infusion, Soxhlet extraction, ultrasound-assisted extraction	HPLC analysis, HPLC-coupled with a diode-array detector and MS-detector	[49,56,57]
Anthocyanins	Cyanidin-glucoside	0.00–23.61 mg/kg [56]; 0.28–0.29 mg/g [49]	Ultrasound-assisted extraction, Soxhlet extraction	HPLC analysis; HPLC-coupled with a diode-array detector and MS-detector	[49,56]
	Cyanidin-arabinoside	0–0.30 mg/g	Ultrasound-Assisted Extraction	HPLC-coupled with a diode-array detector and MS-detector	[49]
	Malvidin 3-O-glucoside	0–1.20 mg/kg	Soxhlet extraction	HPLC analysis	[56]
Triterpenes (µg/g dry weight)	Oleanolic acid	335.20–655.80	Solvent extraction with alkaline hydrolysis	HPLC-coupled with a diode-array detector	[89]
	Ursolic acid	377.00–815.00	Solvent extraction with alkaline hydrolysis	HPLC-coupled with a diode-array detector	[89]

## Data Availability

All data related to this manuscript are available in the form of tables and figures in the manuscript.

## Supplementary Materials

The following supporting information can be downloaded at https://www.mdpi.com/article/10.3390/nu18020350/s1, Table S1: Clinical Trial studies registered on Bilberry.
