# Supplementary material for: A Review of Major Compounds in Bilberry (Vaccinium myrtillus L.) Fruits and Leaves: Isolation, Purification, and Their Antiaging Effects
Source: Nutrients. 2026 Jan 21;18(2):350. doi: 10.3390/nu18020350 (PMC12845149; doi:10.3390/nu18020350)
Supplement: Supplementary file 1 [file nutrients-18-00350-s001.zip › nutrients-3981977-supplementary.pdf]

**Table S1.** Clinical Trial studies registered on Bilberry.

| National Clinical Trial numbers | Year of registration | Title | Description | Country | Status |
|---|---|---|---|---|---|
| NCT06698601 | 2027-04 (estimated) | Impact of Wild Blueberries (*Vaccinium myrtillus*) on the Intestinal Barrier, Microbiome and Inflammation in Chronic Colitis - a Prospective Randomized Crossover Study | The objective of this experimental, prospective, monocentric randomized crossover research is to examine the effect of a blueberry-rich diet related to a blueberry-poor diet on the microbiome, the intestinal barrier, and the inflammatory method in inflammatory bowel disease with chronic colitis. | Germany | Recruiting |
| NCT03620266 | 2025-12-31 (estimated) | Effects of Bilberry and Oat Intake on Plasma Lipid Profile, Inflammation, and Exercise Capacity in Patients With Type 2 Diabetes and/or Myocardial Infarction (BioDiaMI): a Randomized, Double-blind, Placebo-controlled Trial | This is a double-blind, randomized, placebo-controlled clinical trial. The main objective is to evaluate the cardioprotective properties of diet supplementation with dried bilberry and with bioprocessed oat bran, with a secondary explorative objective of evaluating their combination, compared with a neutral isocaloric reference supplement, for patients diagnosed with Type 2 Diabetes Mellitus and Myocardial Infarction. | Sweden | Recruiting |
| NCT06309914 | 2024 | The Effects of Indena's Mirtoselect® Bilberry Extract, Virtiva® Ginkgo Biloba Extract, and Enovita® Grape Seed Extract on Cognitive Performance and Mood States Over a 4-week Time Course | This is a randomized, double-blind, placebo-controlled, 4-week intervention clinical research evaluating the effectiveness of Mirtoselect®, Virtiva® Plus, and Enovita® on cognitive performance and mood states, and the existence of hostile events in response to daily supplementation. | United States | Completed |
| NCT05737108 | 2023 | A Clinical Trial on the Efficacy of Bilberry-containing Capsules for Dry Eye Mitigation | This study examines whether the oral intake of a bilberry capsule product may protect from dry eye symptoms. | Taiwan | Completed |

| | | | | | |
|---|---|---|---|---|---|
| NCT04004182 | 2023 (estimated) | Acute Study of Anthocyanin-rich Bilberry and Polyphenol-rich Apple Extracts on Postprandial Glycaemic Response | This research aims to test the hypothesis that consumption of a fruit bar containing anthocyanin-rich bilberry and polyphenol-rich apple extracts together with a starch and sucrose meal would reduce the postprandial glycemic response. | United Kingdom | Unknown status |
| NCT04380025 | 2021 | The Effects of Mirtogenol® With Bimatoprost on Intraocular Pressure in Hispanics With Open-Angle Glaucoma: A Double-Blind, Randomized Controlled Trial | The objective of this research is to determine if Mirtogenol has an additive effect on the decrease of intraocular pressure when mixed with bimatoprost in the Hispanic population with primary open-angle glaucoma. | Puerto Rico | Unknown status |
| NCT04000139 | 2021 | A Multi-center, Multi-national, Randomized, Double-blind, Placebo Controlled, Parallel Group, Phase IIa Study to Evaluate the Efficacy, Safety, and Tolerability of an Anthocyanin-rich Extract (ACRE) in Patients With Ulcerative Colitis | This research assesses the effectiveness and safety of a bilberry-derived anthocyanin-rich extract in patients with ulcerative colitis. Two-thirds of participants will receive the anthocyanin-rich extract, while one-third will receive a placebo, for 8 weeks of treatment. | Switzerland | Completed |
| NCT04063644 | 2020 | Effect of Visglyc on the Quality of Life of Patients With Dry Eye Symptomatology and Age-related Loss of Visual Acuity | Clinical trial with medical devices, post-authorization, parallel, single-blind, randomized, comparative, prospective to investigate how it affects the quality of life, the use of ocular eye drops on patients with dry eye symptomatology, and age-related loss of visual acuity.<br>Secondary purposes: effects on the dry eye symptomatology, effect on visual acuity, and treatment adherence. | Spain | Completed |
| NCT01180712 | 2019 | Study of Oral Anthocyanins on Insulin Resistance | The objective is to investigate the role of anthocyanin supplementation in the form of a concentrated blaeberry extract on insulin resistance and inflammation, particularly in the adipose tissue following a three-week supplementation period. | United Kingdom | Completed |

| | | | | | |
|---|---|---|---|---|---|
| NCT03213288 | 2018 | The Effects of Bilberry Fruit and Black Rice Derived Anthocyanins on Lipid Status in Adults | The primary objective of the investigation is to directly compare the two major types of anthocyanins found in the diet (cyanidin-type and delphinidin-type) on lipid status in adults. Human intervention trials with anthocyanin-rich berry fruits/extracts such as whole strawberries, elderberry juice, and whortleberry extracts, and purified anthocyanins from a mixture of blackcurrant and bilberry have been shown to beneficially alter biological markers for CVD risk, with beneficial effects on lipid profiles common across these studies. | United Kingdom | Completed |
| NCT03316612 | 2018 (estimated) | The Effect of *Vaccinium myrtillus* L. Extract Intake on Human Metabolism: A Randomized Double-Blind Trial | Advanced glycation end-products (AGEs) have been linked to aging and many metabolic diseases. The findings of previous experiments recommended that the extracts from polyphenol-rich bilberry might inhibit the formation of AGEs. This is a randomized double-blind trial, aims to study the effect of *Vaccinium myrtillus* L. natural extracts on AGEs and human metabolism. | China | Unknown |
| NCT03185676 | 2017 | The Long-Term Efficacy of a Bilberry-Based Probiotic Product on Glucose Tolerance and Insulin Resistance as Compared to a Control. | The influence of a bilberry-based probiotic drink on the postprandial levels of serum glucose and insulin has already been studied in the past. The current study aims to assess the long-term effect of the probiotic bilberry drink on glucose tolerance and insulin resistance in healthy adults and in comparison to a control drink. | Sweden | completed |
| NCT02005796 | 2015 | Bioavailability of Anthocyanins of Blue Potatoes as Compared With Bilberries | In this study, the postprandial glycemia and insulinemia caused by boiled potato with blue-fleshed or yellow-fleshed tubers, or a gel prepared with bilberries and potato starch, are studied in healthy males. Furthermore, the appearance and metabolism of the flavonoids and their metabolites are studied in plasma and urine. | Finland | Completed |

| | | | | | |
|---|---|---|---|---|---|
| NCT01958034 | 2015 | Bilberry as a Dietary Supplement After Myocardial Infarction (The BEAR SMART Trial) | In a double-blinded, randomized, clinical trial of patients suffering from STEMI (ST-segment elevation myocardial infarction) or non-STEMI (non-ST-segment elevation myocardial infarction), compare the effect of 3 months of dietary supplement with bilberry extract on a range of parameters with prognostic importance in cardiovascular disease: lipids, inflammation, oxidative stress, and heart function. | Sweden | completed |
| NCT01245270 | 2013 | A Single Supplement of a Standardised Bilberry Extract (36% (w/w) Anthocyanins) Modifies Glycaemic Response in Persons With Type 2 Diabetes Controlled by Diet and Lifestyle. | This study validates that the incorporation of a concentrated bilberry extract decreases the postprandial glycaemia and insulin in volunteers with type 2 diabetes. The most likely mode of action for the lower glycaemic effects includes a reduction in rates of carbohydrate digestion and/or absorption. | United Kingdom | Completed |
| NCT01414647 | 2010 | The Effect of a Diet Rich in Nordic Berries on Gut Microbiota, Glucose and Lipid Metabolism, and Metabolism of Fenolic Compounds | Dietary polyphenols might have beneficial effects on glucose and lipid metabolism based on the studies made in animals or cell cultures. The findings regarding the possible reduction of low-grade inflammation are also prevailing in humans. Low-grade inflammation has been recommended to be a mechanistic link between obesity and its significance on cardiometabolic health. The current research aims to inspect the effect of a diet rich in berries on glucose and lipid metabolism and inflammatory markers. | Finland | Completed |
| NCT00972972 | 2009 | Effect of Bilberry (European Blueberries) and Grape Polyphenols on Cognition and Blood Parameters in Normal, Aged Men With Subjective Memory Decline | The purpose of this investigation is to find out whether dietary intervention with blueberry and grape juice extracts in elderly men with subjective memory problems would raise performance on neuropsychological memory tests and change biomarkers of muscle damage and whole blood gene expression profiles. | Norway | Unknown status |

| | | | | | |
|---|---|---|---|---|---|
| NCT01860547 | 2009 | The Effect of the Bioactives of Sea Buckthorn and Bilberry on the Risk of Metabolic Diseases | The study hypothesis is that the bioactive compounds of sea buckthorn berries (*Hippophae rhamnoides*), their fractions, and bilberries (*Vaccinium myrtillus*) have positive effects on lipid and carbohydrate metabolism and will thus reduce the risk of developing metabolic diseases. | Finland | Completed |
| NCT02194361 | 2000 | Efficacy of a Bilberry Extract Standardised to a Content of 25% Anthocyanosides in Improving the Night Vision of Healthy Volunteers: a Double-blind, Randomized, Placebo-controlled, Cross-over Trial Over 2 x 28 Days | Study to investigate the efficacy of standardized bilberry extract in enhancing night vision and to estimate its tolerability and safety. | Not provided | completed |
| NCT02194127 | 1999 | Efficacy and Tolerability of a Treatment Over 28 Days With a Bilberry Extract Standardized to a Content of 25% Anthocyanidines in Volunteers With Impaired Twilight and Night Vision | Study to find out the efficiency of anthocyanin to improve impaired twilight and night vision and to test its tolerability and safety. | Location not provided | Completed |

Source: https://clinicaltrials.gov/search?cond=bilberry.
